# Highly Nonrandom Features of Synaptic Connectivity in Local Cortical Circuits

**DOI:** 10.1371/journal.pbio.0030068

**Published:** 2005-03-01

**Authors:** Sen Song, Per Jesper Sjöström, Markus Reigl, Sacha Nelson, Dmitri B Chklovskii

**Affiliations:** **1**Cold Spring Harbor Laboratory, Cold Spring HarborNew YorkUnited States of America; **2**Department of Biology and Volen National Center for Complex Systems, Brandeis UniversityWaltham, MassachusettsUnited States of America; **3**Wolfson Institute for Biomedical Research and Department of Physiology, University CollegeLondonUnited Kingdom; University College LondonUnited Kingdom

## Abstract

How different is local cortical circuitry from a random network? To answer this question, we probed synaptic connections with several hundred simultaneous quadruple whole-cell recordings from layer 5 pyramidal neurons in the rat visual cortex. Analysis of this dataset revealed several nonrandom features in synaptic connectivity. We confirmed previous reports that bidirectional connections are more common than expected in a random network. We found that several highly clustered three-neuron connectivity patterns are overrepresented, suggesting that connections tend to cluster together. We also analyzed synaptic connection strength as defined by the peak excitatory postsynaptic potential amplitude. We found that the distribution of synaptic connection strength differs significantly from the Poisson distribution and can be fitted by a lognormal distribution. Such a distribution has a heavier tail and implies that synaptic weight is concentrated among few synaptic connections. In addition, the strengths of synaptic connections sharing pre- or postsynaptic neurons are correlated, implying that strong connections are even more clustered than the weak ones. Therefore, the local cortical network structure can be viewed as a skeleton of stronger connections in a sea of weaker ones. Such a skeleton is likely to play an important role in network dynamics and should be investigated further.

## Abstract 2

A dataset of hundreds of recordings in which four neurons were simultaneously monitored reveals clustered connectivity patterns among cortical neurons

## Introduction

Understanding cortical function requires unraveling synaptic connectivity in cortical circuits, that is, establishing the wiring diagrams. Although smaller wiring diagrams can be reconstructed using electron microscopy [1], reconstruction on the scale of a cortical column is impossible with current technology (but see [2] for a promising approach). Even if such a possibility were within reach, synaptic connectivity likely varies from animal to animal and within one animal over time. Therefore, a reasonable approach is to describe synaptic connectivity statistically, or probabilistically. Such statistical description may be based on random sampling of connections with multineuron recordings [3,4,5]. For example, electrophysiological recordings from neuronal pairs showed that the probability of connection is often low [3,5,6,7,8,9,10,11], suggesting that the network is sparse. Such statistical approaches may create the impression that synaptic connectivity in local cortical circuits is random. This view is consistent with previous suggestions [12,13,14], but hard to reconcile with cortical functionality, which must rely on specificity of connections [15,16,17,18].

In general, statistical sampling of connections does not imply that the underlying network has random connectivity. Indeed, statistical sampling has already revealed several nonrandom features in cortical connectivity. In particular, specific connectivity patterns exist between different classes in the local circuit [3,19,20]. Within one putatively homogeneous class, the number of reciprocally connected pairs is greater than expected in a random network [5,6,11]. Distribution of the number of synapses per connection is non-Poisson and has low variance [6,21]. At the same time, the distribution of the connection strength is broad [5,6,10,11]. But many questions remain unanswered: Are there nonrandom features in patterns involving more than two neurons? What is the distribution of synaptic connection strength? Are synaptic connection strengths correlated?

Recently, new approaches for network connectivity analysis have been developed and various nonrandom features were discovered in natural and man-made networks. In particular, many networks are scale-free, that is, the number of connections per node (node degree) often follows a power-law distribution [22]. Also, many networks exhibit the small-world property, that is, high local clustering of connections in combination with a short path between any two nodes [23,24]. In addition, probability of connection between nodes depends on how many connections they have [25]. Although local cortical networks may possess these properties, existing connectivity data are not sufficient for such analyses. These data are obtained by random sampling of connections and call for other approaches. One such approach is to explore local structures in network connectivity by studying the distribution of few-node connectivity patterns, or motifs [26,27]. Another such approach is analyzing the utilization (or, in this case, the strength) of connections [28,29,30].

In this paper, we apply a combination of statistical methods to a large dataset from hundreds of simultaneous quadruple whole-cell recordings from visual cortex in developing rats. Our results confirm previous indications of nonrandomness and point out several new ones. In particular, we show that the distribution of connection strengths between pyramidal neurons is non-Poisson and find correlations in the strength of the connections sharing pre- or postsynaptic neurons. Also, we find several overrepresented three-neuron connectivity patterns, or motifs. Surprisingly, we find that some few-neuron motifs can play an important role in the dynamics of layer 5 local cortical networks because they are composed of exceptionally strong connections. This suggests a novel view of the local cortical network, in which a skeleton of stronger connections is immersed in a sea of weaker ones.

## Results

**Figure 1:**
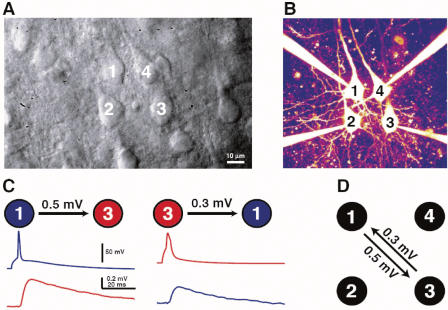
Illustration of a Quadruple Whole-Cell Recording (A) Dodt contrast image showing four thick-tufted L5 neurons before patching on. (B) Fluorescent image of the same four cells in whole-cell configuration. (C) Average EPSP waveform measured in the postsynaptic neuron (bottom) while evoking action potentials in the presynaptic neuron (top). (D) Diagram of detected synaptic connections and their strengths for this quadruple recording.

We studied connectivity among thick tufted layer 5 neurons in rat visual cortex with quadruple whole-cell recordings (Figure 1A and 1B). Thick tufted layer 5 pyramidal neurons are important projection neurons from the cerebral cortex to subcortical areas [9,31,32]. Synaptic connection strengths were assessed by evoking action potentials in each of the four cells and measuring the averaged peak excitatory postsynaptic potential (EPSP) amplitudes in the other three cells (see Figure 1C and Materials and Methods). Results of these measurements for a sample quadruple recording are shown in Figure 1D. Each arrow indicates a detected connection with the corresponding connection strengths. The dataset contained a total of 816 quadruple recording attempts (some of these attempts contained data for only two or three neurons, if whole-cell configuration was not successfully established with all four cells). As previously reported [5], the rate of connectivity was *p* = 11.6% (931 connections out of 8,050 possible connections), which is similar to that reported for rat somatosensory cortex layers 5 [6,9] and 2/3 [11], as well as those previously reported for rat visual cortical layers 5 [3,10] and 2/3 [11].

### Two-Neuron Patterns

**Figure 2:**
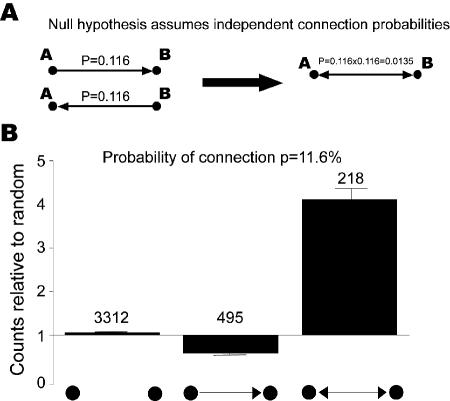
Two-Neuron Connectivity Patterns Are Nonrandom (A) Null hypothesis is generated by assuming independent probabilities of connection. (B) Reciprocal connections are four times more likely than predicted by the null hypothesis (*p* < 0.0001, Monte Carlo simulation to test for overrepresentation). Numbers on top of bars are actual counts. Error bars are standard deviations estimated by bootstrap method.

We started by assessing how well a randomly connected network [33] describes our dataset. In this model, the existence of a connection between any two neurons is independently chosen with a uniform probability *p* (Figure 2A). We test the predictions of this model by classifying all simultaneously recorded pairs of neurons into three classes: unconnected, unidirectionally connected, and bidirectionally connected. Given connection probability *p* and total number of pairs *N,* the expected number of unconnected pairs should be *N(1 − p)* ^2^. The expected number of unidirectionally connected pairs should be *2Np(1 − p),* and the expected number of bidirectionally connected pairs should be *Np^2^.* We find that the actual number of bidirectionally connected pairs is four times that of the expected numbers (*p* < 0.0001) (Figure 2B). The observed overrepresentation of reciprocally connected layer 5 neurons confirms previous reports [5,6]. Such overrepresentation has also been observed in layer 2/3 of the rat visual cortex [11]. However, whether projections between layers observe this pattern remains an open question.

**Figure 3:**
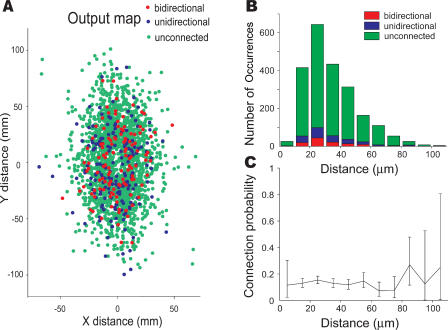
Probability of Connection among Adjacent Neurons Does Not Depend Strongly on the Interneuron Distance (A) Relative location of labeled neurons in the plane of the section. Positive direction of y-axis is aligned with apical dendrite. Potentially presynaptic neuron is located at the origin. Red—bidirectionally connected pairs; blue—unidirectionally connected pairs; green—unconnected pairs. (B) Histogram showing the numbers of pairs in the three classes as a function of distance between neurons (Euclidian distance was calculated from relative *X, Y, Z* coordinates). (C) Probability of connection versus interneuron distance. Error bars are 95% confidence intervals estimated from binomial distribution.

Can the overrepresentation of reciprocal connections reflect an experimental artifact? Indeed, such overrepresentation could arise from a nonuniform probability of connection for different neurons. For example, connection probability may depend on interneuron distance. To control for this artifact, we measured distances between neurons in recordings where labeling was performed successfully. We found that the probability of connection does not depend systematically on the interneuron distance (*p* = 0.21, chi square test) (Figures 3, S1, and S2). This is not surprising because most neurons were located closer than the span of their dendritic (and especially axonal) arbors. Our result is consistent with Holmgren et al.'s study [11], which found only a small (22%) decrease in connection probability up to 50 μm, with a more significant drop (44%) up to 100 μm for layer 2/3 pyramidal neurons.

Another source of nonuniformity in connection probability may be axonal arbors being cut off differentially, depending on the depth of the recorded neuron from the slice surface. (The recording depths were from 10 to 100 μm.) To explore such a possibility we measured the dependence of the connection probability on the recording depth. Neither connection probability, nor strength of connection was found to depend systematically on the recording depth (see Figure S3). In addition, for every successfully labeled tissue we measured the distance from the average cell position to the nearest axonal cut point (see Figure S3). Again no strong trends in connection probability or connection strength were found. These results show that the cutting artifact is unlikely to explain observed nonrandom features.

We also considered the possible artifact of connection probability varying with age. We found a weak decline in connection probability and EPSP amplitude (consistent with Reyes and Sakmann [34]) within the range used in experiments (P12–P20; see Figure S4). Yet, such a decline is insufficient to account for the observed nonrandomness. To demonstrate this, we repeated most of the analysis on a subset of the data from 14 to 16-d-old animals when the majority of measurements were performed (see Figure S5). We found that bidirectional connections are also overrepresented in this subset of data. Results of other analyses that will be described later in the paper are also confirmed on this subset (Figure S5).

Finally, it is possible that some extreme degree of inhomogeneity in connections probability is able to explain the observed overrepresentation of reciprocal pairs, but this would probably reflect large local inhomogeneity in cortical connectivity patterns—possibly differences between subclasses [6,35], rather than experimental artifacts—and is in line with the main conclusions of this paper.

### Three-Neuron Patterns

**Figure 4:**
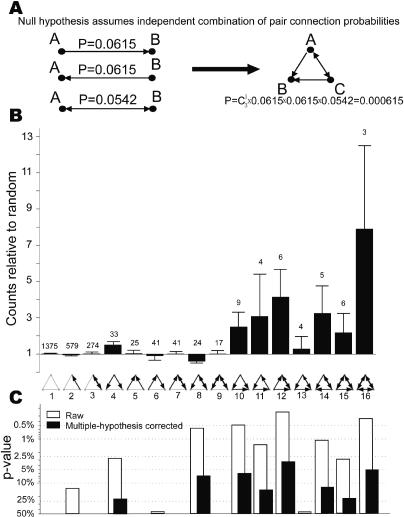
Several Three-Neuron Patterns Are Overrepresented as Compared to the Random Network (A) Null hypothesis for three-neuron patterns assumes independent combinations of connection probabilities of two kinds of two-neuron patterns. (B) Ratio of actual counts (numbers above bars) to that predicted by the null hypothesis. Error bars are standard deviations estimated by bootstrap method. (C) Raw (open bars) and multiple-hypothesis testing corrected (filled bars) *p*-values. *p*-values above 0.5 are not shown.

We extended our analysis by comparing the statistics of three-neuron patterns to those expected by chance [26,27]. We classify all triplets into 16 classes and count the number of triplets in each class. In order to avoid reporting overrepresented three-neuron patterns just because they contain popular two-neuron patterns, we have revised the null hypothesis[26,27]. The control distribution was obtained numerically by constructing random triplets where constituent pairs are chosen independently, but with the same probability of bidirectional and unidirectional connections as in data (Figure 4A). For example, the actual probability of a unidirectional connection is (according to Figure 2B) 495/(3312 + 495 + 218) = 0.123. Then the probability of unidirectional connection from A to B is 0.123/2 = 0.0615, the same as from B to A (see Figure 4A). The probability of bidirectional connection is (according to Figure 2B) 218/(3312 + 495 + 218) = 0.0542. The probability of finding the particular triplet class in Figure 4A by chance is the product of the probabilities of finding the three constituent pairs and a factor to account for permutations of the three neurons. The ratio of the observed counts and the expected counts for each class are plotted in Figure 4B. The actual counts are given as numbers on top of the bars. Although triplets from several of these patterns have been reported previously [9,10], small numbers of recordings have precluded statistical analysis.

According to Figure 4B, several triplet patterns are overrepresented. Is this overrepresentation statistically significant? Because we look for overrepresentation in many pattern classes at the same time, the raw *p*-values (Figure 4C) overestimate the true significance (underestimate the true *p*-value). To correct the raw *p*-values, one has to apply a multiple-hypothesis testing procedure. We chose to correct the *p*-values by applying a step-down min-P-based multiple-hypothesis testing correction procedure (see Materials and Methods). The corrected *p*-values (Figure 4C) give the probability of mistakenly reporting at least one of the patterns as overrepresented when it is not.

Two-neuron correlations are summarized by saying that if neuron A synapses onto neuron B, then the probability of B synapsing onto A is several times greater than chance. Three-neuron correlations are summarized roughly by saying that if A connects with B and B connects with C (regardless of direction), the probability of connection between A and C is several times greater than chance. Interestingly, similar results have been obtained in the analysis of the Caenorhabditis elegans wiring diagram [36], which was reconstructed from serial section electron microscopy [1]. Because different techniques have different biases, the similarity of results suggests that correlations in synaptic connectivity represent a general property of neuronal circuits. Such property may represent evolutionary conservation from invertebrates to mammals or convergence driven by similar computational constraints.

Although individual connectivity patterns containing more than three neurons could not be analyzed statistically for the existing dataset (Table S1), we found a 70% overrepresentation of “chain” quadruplets (patterns number 21 23 24 26 28 29 31 32 33 34 35 38 39 41 43 as defined in Figure S6, *p* = 0.004) relative to the null hypothesis requiring that a random matrix has the actual proportion of triplet classes.

### Distribution of Synaptic Connection Strengths

**Figure 5:**
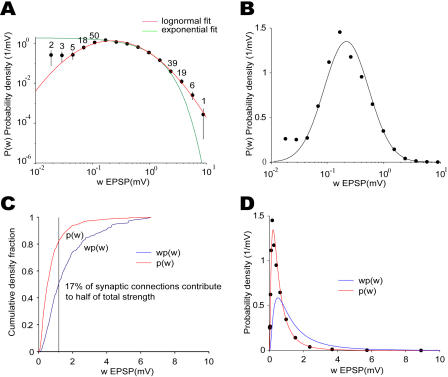
Distribution of Synaptic Connection Strength Has a Heavy Tail (A) Estimated probability density function in log–log space, with both lognormal fit (*p*[*w*] = 0.426exp[−(ln[*w*] + 0.702)^2^/(2 × 0.9355)^2^]/*w*) and exponential fit (*p*[*w*] = 1.82exp[−1.683*w*]). Notice that the lognormal fit has a heavier tail than the exponential distribution. Error bars are standard deviations estimated by bootstrap method (not shown when narrower than the dot). The numbers on top on the dots are the actual counts (not shown when more than 50). (B) Estimated probability density distribution in semilog space, with the lognormal fit. The lognormal function shows up as a normal function in the semilog space. (C) Empirical cumulative density function for both the probability distribution of synaptic strengths and the synaptic contribution (normalized product of probability and connection strength). They are generated directly from the data rather than the fits. The vertical line illustrates the fact that 17% of the synaptic connections contribute to half of the total synaptic strengths. (D) Probability density function of synaptic connection strengths p(w) fitted by a lognormal function and the synaptic contribution defined as the product of the strength, w, and p(w). The total areas under both curves are normalized to 1.

Next, we turned our attention to the distribution of synaptic connection strengths as characterized by EPSP amplitude (Figure 5A). We estimated the probability density function by binning connection strengths and dividing the number of occurrences in each bin by the bin size. Since there are many more weak connections than strong ones, we chose bins whose sizes increase linearly with the connection strength at the bin center. In other words, bin sizes are uniform on the log scale. The estimated density function is independent of the chosen bin size since the bin size is divided out.

The obtained distribution has a mean of 0.77 mV and a heavy tail, that is, a greater number of strong synaptic connections than expected for either the exponential distribution (Figure 5A) or the normal distribution (not shown). There are significantly more connections with strengths above 1 mV than expected by best exponential or normal fit (*p* < 0.0001; see Materials and Methods). We find that the dataset is best fit by a lognormal distribution, which has a bell shape when plotted on a semilog scale (Figure 5B). Although our measurements were performed in developing animals, experiments in mature animals have also revealed large single EPSPs (>5 mV) [37].

The overrepresentation of strong synaptic connections is likely to have important implications for the cortical network dynamics. This is because strong connections are few but powerful. For example, although synaptic connections with strength above 1.2 mV constitute only 17% of all connections, they contribute about half of the total synaptic weight (Figure 5C and 5D). This estimate was obtained by multiplying the number of synaptic connections by the connection strengths (assuming equal presynaptic firing rates).

### Correlation of Connection Strengths in Two-Neuron Patterns

**Figure 6:**
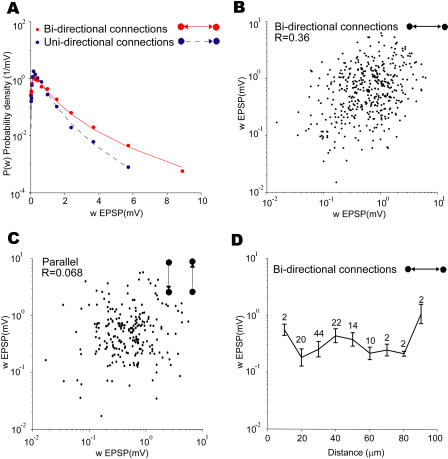
Bidirectionally Connected Pairs Contain Connections That Are Stronger and Correlated (A) Synaptic connections in bidirectionally connected pairs are on average stronger than those in unidirectionally connected pairs. The probability density distribution for both the reciprocal (red solid, p(w) = 0.41exp(−(ln w + 0.60)^2^/(2 × 0.976^2^)/w) and nonreciprocal (blue dashed, p(w) = 0.47exp(−(ln w + 0.81)^2^/(2 × 0.834^2^)/w) connections are shown. (B) In bidirectionally connected pairs synaptic connection strengths are moderately but significantly correlated (*R* = 0.36, *p* < 0.0001). (C) Scatter plot of the strength of synaptic connections that shared no pre- and postsynaptic neurons in the same quadruple recording. There might be other connections in the quadruplet besides these two connections. No significant correlation is observed (*R* = 0.068, *p* = 0.48). All correlations calculated using Pearson's *R* method in log space. (D) Average connection strength for bidirectional connections does not vary systemically with interneuron distance (one-way ANOVA, *p* = 0.068). Numbers on top of data points are the number of connections. Error bars are standard errors of the mean.

Next, we analyzed the correlations between the strengths of the synaptic connections in two-neuron patterns. We find that the synaptic strengths of the bidirectional connections are on average stronger than the unidirectional synaptic connections (mean 0.95 mV versus 0.61 mV, *p* = 3.1 × 10^−7^, Student's *t*-test) in agreement with [6]. The distribution of connection strengths for the bidirectional connections is expanded toward stronger connections compared to that of unidirectional connections (Figure 6A; note the semilog scale). Furthermore, the strengths of the two connections in a bidirectional pair are moderately but significantly correlated with each other (Figure 6B). To control for possible systematic variations between different quadruplets, we looked at correlations in the strength of synaptic connections that shared no pre- and postsynaptic neurons and found no significant correlation (Figure 6C). Could the correlation in connection strength result from nearby neurons having stronger connections? We do not think so because the strengths of bidirectional connections do not depend strongly on the distance between neurons (Figure 6D).

**Figure 7:**
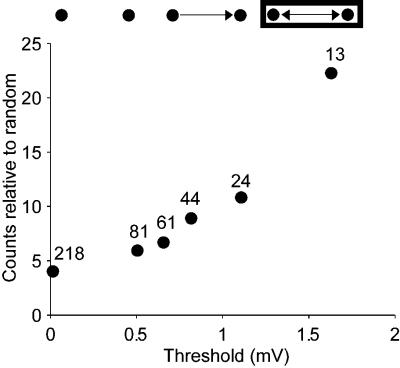
Stronger Connections Are More Likely Reciprocal than Weaker Ones Overrepresentation of bidirectionally connected motifs gets more dramatic for higher threshold of connection strength (counts differ from random with *p* < 0.001 for all thresholds, Monte Carlo simulation). Significance of monotonicity is assessed by applying the Kolmogorov-Smirnov test (*p* < 3.5 × 10^−10^ for all successive pairs). Numbers on top of dots show the counts of actual pairs.

Another way to characterize correlations in connection strengths is by analyzing the overrepresentation of the bidirectional motif for different synaptic-strength thresholds. For every threshold value, we modify the dataset by keeping only those synaptic connections that exceed this threshold. In a unidirectionally connected pair, connection is kept only if its strength exceeds the threshold. In a bidirectionally connected pair, if both connections exceed the threshold, they are both kept. If only one of the connections exceeds the threshold, the pair becomes unidirectionally connected. Then we predict the numbers of bidirectional synaptic connections that exceed threshold by using the null model assuming independent probability, as was done for two-neuron patterns. The actual number of bidirectional connections exceeding the threshold is compared with the predicted. We find that, as the threshold is raised, the ratio of actual to expected number of bidirectional connections monotonically increases (Figure 7). This shows that reciprocity of connections is greater for stronger connections.

### Three-Neuron Patterns with Strong Connections

**Figure 8:**
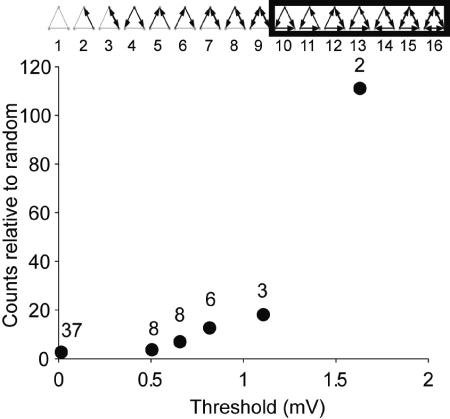
Stronger Connections Are More Clustered than Weaker Ones Relative overrepresentation of highly connected three-neuron motifs monotonically increases as the threshold is raised (counts differ from random *p* < 0.001 for all thresholds, Monte Carlo simulation). Significance of monotonicity is assessed by applying the Kolmogorov-Smirnov test (*p* <3.5 × 10^−10^ for all successive pairs). Numbers show the actual triplet counts. For the second to highest threshold, two instances of pattern 12 and one instance of pattern 16 survive. For the highest threshold, one instance each of pattern 12 and 15 survive (one of the connections in pattern 16 drops out and it becomes pattern 15).

We also analyzed the overrepresentation of three-neuron patterns as a function of threshold. Because of small numbers of patterns in some classes, we have grouped highly connected patterns (boxed patterns in Figure 8) together and calculated the measured counts relative to random for different thresholds. Similar to the two-neuron motifs, overrepresentation of the highly connected motifs gets more dramatic as the threshold is raised (Figure 8). Although the numbers of overrepresented three-neuron patterns are small, they may contribute to the neuronal dynamics in nontrivial ways, for example, by supporting recurrent activity. Furthermore, the contribution of three-neuron patterns depends on the chosen connection strength threshold. The relative fraction of overrepresented patterns in the network of stronger connections is much greater than that in the network of weaker connections.

In addition, we analyzed the strengths of synaptic connections made onto the same neuron, synaptic connections coming out of the same neuron, and synaptic connections onto and out of the same neuron (Figure S7). These strengths are weakly correlated. Correlations in the strength of incoming or outgoing connections may suggest, although not conclusively prove, the presence of neurons with particularly strong connections. Such neurons may be analogous to “network hubs,” or nodes with particularly large numbers of connections (degrees), which are known to exist in other networks [22,38].

## Discussion

**Figure 9:**
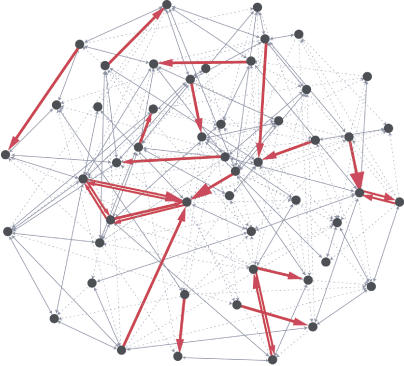
Statistically Reconstructed Network of 50 Layer 5 Pyramidal Neurons Illustrates That Stronger Connections form a Skeleton Immersed in a Sea of the Weaker Ones Details of statistical reconstruction are given in Materials and Methods. For illustrative purposes, neurons are arranged so that strongly interconnected nodes are close by. Dotted arrows are weak (<1 mV) unidirectional connections; solid arrows are weak bidirectional connections. Red arrows are strong (>1 mV) unidirectional connections with arrow size indicating the strength. Red arrows with double lines are strong bidirectional connections.

We showed that synaptic connectivity in the local network of layer 5 pyramidal neurons is highly nonrandom. The network consists of sparse synaptic connections that tend to cluster together in the form of overrepresented patterns, or motifs. The distribution of connection strengths has a significant tail; strong connections are few but powerful and even more clustered than the weak ones. These results suggest that the network may be viewed as a skeleton of stronger connections in a sea of weaker ones (Figure 9). Interestingly, the existence of few but powerful synaptic connections makes analyzing the network with few-neuron connectivity patterns a reasonable first step. Indeed one could have thought that, since each neuron receives inputs from thousands of others collectively determining its dynamics, analysis of few-neuron motifs is akin to “searching under the street light.” Yet, the finding of a heavy tail in the connection strength distribution suggests that a lot of power is due to a few connections. Therefore, our analysis has illuminated a significant part of the local cortical architecture, especially if the stronger connections are distributed uniformly among neurons. Naturally, this description is not complete, and future studies should investigate whether stronger synaptic connections are distributed among neurons uniformly or belong preferentially to “hub” neurons. Also, studies involving larger networks of neurons will be needed to provide a more complete understanding of the network structure and function.

Although broad distribution of synaptic connections strength has been seen in the cortex [6,11] and in the cerebellum [39], heavy-tailed distributions have not been suggested as suitable fits previously. For example, in the feed-forward projection from granule to Purkinje cells in the cerebellum, the distribution was fitted by a truncated Gaussian distribution, argued to be optimal for information storage [40]. It would be interesting to see if analogous theory could be developed to explain the lognormal distribution seen among the layer 5 pyramid recurrent connections. Another relevant observation is that of mini-EPSC amplitudes [41], which were fitted by a Poisson distribution based on a binomial model of the data. In this case, however, we are looking at direct unitary connections between pairs of neurons rather than individual synapses, and such direct connections between nearby cortical neurons are typically comprised of multiple individual synapses [6,21,34,42]. Evoked and spontaneous release may also produce different synaptic strength distributions because the underlying molecular mechanisms are different. Alternatively, the lognormal distribution could depend on network activity patterns not present in dissociated cultures.

To illustrate the possible impact of the skeleton of strong connections on the network dynamics, let us consider a local network of layer 5 neurons occupying the 300 μm × 300 μm area. According to Peters et al. [43], there are 4,480 thick tufted layer 5 neurons under 1 mm^2^ of cortex, and therefore 400 thick tufted neurons in the considered network. With a connection probability of 0.11, each neuron receives inputs from 44 others. If distribution of connection strength is uniform among neurons, then each neuron has only about 2–3 connections in the greater than 2-mV range. If the corresponding 2–3 presynaptic neurons fire simultaneously, they may drive the postsynaptic neuron to fire. This suggests that a sparse skeleton of strong connections may drive the dynamics of the network. An exceptionally strong connection (>10 mV*)* may alone drive a postsynaptic neuron to fire. Suprathreshold EPSPs have been observed previously with paired recordings [37,44,45] and with calcium imaging [46]. However, such connections occur with a very low probability (about 1/1000, estimated from lognormal distribution), meaning that there are only about 20 of such connections in the considered network and that therefore most neurons do not have them. Finally, inhibitory neurons may make it more difficult to drive a postsynaptic neuron to fire and need to be investigated.

Because the highly influential, strong, and reliable (Figure S8) synaptic connections in the network are few in number, their exact connectivity pattern and properties might therefore be important and make firing patterns of the involved cortical neurons highly reproducible. This may be manifested in the simultaneous activation of several neurons in organized patterns during spontaneous and evoked activity that has been observed in cortical slices [47,48,49,50] and elsewhere [51,52,53,54]. Unfortunately, most current experimental studies rely on random sampling of neurons appropriate for studying the properties of average connections rather than the particularly strong connections. It might be important in the future to devise methods to selectively study particularly strong connections, because of their anticipated large influence on network dynamics [35,49].

Although stronger connections are likely to be important for network dynamics, weaker connections need to be considered as well. Collectively, they could affect the dynamics of the network significantly and might carry out computations with a population code. The weaker connections may be a potent driving force if firing is correlated between neurons. In addition, weaker connections may serve as a potential reserve for cortical plasticity. Indeed, weaker connections could be strengthened easily through a variety of activity-dependent learning rules (see below for an example).

In neurobiological literature, synaptic connections have been classified previously by their impact into “drivers” and “modulators” [55]. Drivers are less numerous and produce stronger impact than modulators. We stopped short of calling stronger connections among layer 5 pyramidal neurons drivers, and weaker connections modulators, for two reasons. First, previously drivers and modulators have been used to describe inputs arising from a priori different subsets of neurons, such as different pathways. Second, we do not find a clearly bimodal distribution of connections strength, suggesting that the distinction between stronger and weaker connections is not clearly defined enough to warrant two separate classes.

Next, we consider how observed distributions of synaptic strength and correlations between them might have arisen. Although it is possible that the neurons bound by stronger connections form a distinct subclass defined by perhaps distinct long-range projection patterns, or different channel densities and/or gene expression patterns, it is also possible that the distributions arise as a natural consequence of activity-dependent plasticity rules.

The lognormal distribution of synaptic connection strength may be explained by a random multiplicative process, which has been extensively studied previously [56,57,58,59]. The idea behind this is demonstrated below. Suppose a synaptic connection changes its strength multiplicatively after *i*th plasticity episode. This can be expressed as


*w*_i_ = *F*_i_*w*_i-1_ ,

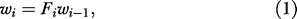



where *w_i_* is the synaptic connection strength after *i*th plasticity episode, and *F*_i_ is the fractional change in synaptic strength induced by that episode. Then it is easy to see that



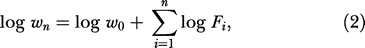



where *w_n_* is the current synaptic connection strength, *w*_0_ is the initial synaptic connection strength, and *F_i_*s are the fractional changes in synaptic connection strength. If we assume all *F_i_*s to be independent and identically distributed with finite mean and variance, then by applying central limit theorem, log *w_n_* should obey a Gaussian distribution, which implies that *w_n_* obeys a lognormal distribution. For a more rigorous treatment, a decay term has to be added to make the distribution stationary, which is analogous to the Gompertz stochastic growth model in ecology [56,59] (also see Appendix S1). In a network, the fractional change in synaptic connection strengths due to long-term potentiation (LTP) would have complex dependencies both on current synaptic connection strength and on correlated activity in the network. In a previous study of layer 5 pyramidal cells, we found only a weak—although statistically significant—dependence of the percentage amount of LTP on the pre-LTP EPSP amplitude so that fractional synaptic change due to LTP was in effect approximately constant for most synaptic strengths [5]. Studies in other brain areas have found a more marked negative dependency [60,61,62]. However, this negative dependency could be counterbalanced by the stronger correlation between presynaptic and postsynaptic firing patterns introduced by a stronger synaptic connection. Regardless, it is curious that a simple independency assumption, together with synaptic decay, reproduces the observed distribution, despite the complex interactions in the network. How this is achieved warrants further investigation.

Can the overrepresentation of bidirectional connections and the correlation in the reciprocal connection strength arise from known learning rules? For example, the synaptic connections studied in this paper are known to obey a temporally asymmetric spike-timing-dependent plasticity rule [5,8], in which the strength of a synaptic connection changes according to the timing of pre- and postsynaptic spikes. If a presynaptic spike shortly precedes a postsynaptic spike, the synaptic connection is strengthened. Conversely, if a presynaptic spike follows a postsynaptic spike, the synaptic connection is weakened. Simulations have shown that in a recurrent network, if effects of spike pairs are assumed to sum linearly, this rule leads to underrepresentation of bidirectional motifs, instead of the overrepresentation observed here [63], because the firing statistics are exactly reversed for the reciprocal synaptic connections. However, for highly correlated firing of pre- and postsynaptic cells, depending on the relative durations and amplitudes of the long-term potentiation and long-term depression temporal windows, more potentiation than depression may be triggered for connections going both ways [64,65]. Furthermore, nonlinear spike interactions are known to operate at those synapses. In particular, the spike-timing-dependent plasticity rule becomes temporally symmetric if the pre- and postsynaptic neurons fire at higher than 50 Hz [5]. Whether these factors can explain this discrepancy, or additional factors need to be considered, remains to be studied.

In a wide range of networks, there is a power-law relationship between the numbers of connections a particular node has (its degree) and the abundance of such nodes [66]. These networks have been termed scale-free networks [22]. In particular, such a power-law distribution of the number of connections a neuron makes has been reported in C. elegans [22]. Here, we have not studied the degree distribution because of the lack of adequate data (such as, for example, the full connectivity diagram for the cortical network). We instead analyzed the strengths of the connections and found a lognormal distribution of synaptic connection strengths, which has a heavy tail, similar to the power-law distribution. Similar distributions have been observed in many nonbiological networks [67,68]. In the biological setting, using an in silico model of metabolic flow in yeast, Almaas et al. [28] found that network use is highly uneven and dominated by several “hot links” that represent high-activity interactions that are embedded into a web of less active interactions. Such heavy-tailed distribution for connection strengths has also been suggested based on experimental data for metabolic flow and gene regulation networks [29,30]. Therefore, a heavy-tailed distribution for connection strengths along with clustering of stronger connections into a backbone might represent a novel universal feature of many networks, in addition to the power-law distribution of number of connections commonly discussed. Such an arrangement would give the stronger links a larger role in the network and might represent a hierarchal organizational scheme of the network structure [38].

In conclusion, the statistics of connectivity in a local network of layer 5 tufted pyramidal neurons are highly nonrandom and bear similarities to other biological networks. The cortical network is best visualized as a skeleton of stronger connections in a sea of weaker connections. These findings are likely to have important implications for cortical dynamics.

## Materials and Methods

### 

#### Electrophysiology

The dataset used for this study was originally used for the study of long-term plasticity, and the methods were previously described in detail [5,69]. Briefly, acute visual cortical slices were cut from rats aged P12–P20. Rats were anesthetized with isoflurane, decapitated, and the brain was rapidly removed to ice-cold artificial cerebrospinal fluid (in mM: NaCl, 126; KCl, 3; MgCl_2_, 1; NaH_2_PO4, 1; CaCl_2_, 2.5; NaHCO_3_, 25; dextrose, 25; osmolality 320 mOsm, bubbled with 95% O_2_/5% CO_2_ [pH 7.4]). Slices were used after at least 1 h of incubation, and up to 11 h after slicing. Recordings were done at 32–34 °C.

Whole-cell recording pipettes (5–10 MΩ, 1–2 μm diameter) were filled with (in mM): KCl, 20; (K)Gluconate, 100; (K)HEPES, 10; (Mg)ATP, 4; (Na)GTP, 0.3; (Na)Phosphocreatine, 10; and 0.1% w/v biocytin, adjusted with KOH to pH 7.4, and with sucrose to 290–300 mOsm. Thick tufted L5 neurons were identified at 400X magnification using IR-DIC optics (Olympus BX-50; Olympus, Melville, New York, United States). To ensure that arborizations of recorded L5 neurons were minimally damaged during dissection, slices were used only if L5 apical dendrites were approximately parallel with the slice surface and could be traced most or all of the way to the pial surface. Gigaohm seals were then established on four neurons, after which breakthroughs were performed in quick succession. In some cases, one or two breakthroughs failed, thus yielding triple or double recordings; connections found in these cases were included in the dataset. Signals were amplified with AxoPatch 200B, AxoPatch-1B, and AxoClamp 2B amplifiers (Axon Instruments, Foster City, California, United States) and filtered at 5 kHz. Acquisition was done at 10 kHz using MIO-16E boards (National Instruments, Austin, Texas, United States) and custom software running on Igor Pro (WaveMetrics, Lake Oswego, Oregon, United States) on Macintosh computers (Apple Computer, Cupertino, California, United States). Recordings were terminated if membrane potential changed more than 8 mV or input resistance (measured from 250-ms-long 25 pA hyperpolarizing pulses preceding each trace) changed more than 30% from the baseline.

#### Measurement of synaptic connection strengths

We assessed connectivity by averaging ten or more traces. Synaptic connection strength was calculated by averaging the peak EPSP amplitudes (measured using a 1-ms-long window centered on the peak of the averaged EPSP trace) from 45 to 60 responses obtained during a 10- to 15-min-long baseline period just after breakthrough. In some cases (less than 5%), the EPSP amplitude was determined from fewer than 45 responses (although never fewer than 10 responses), typically because the recordings failed. The standard deviation of EPSP amplitude is within 0.04–1.4 mV and depends weakly on the mean EPSP amplitude (see Figure S8). As the averaged EPSP waveforms were time-locked to the presynaptic spike, the signal-to-noise ratio was good enough to allow for detection of synaptic connections with strengths as low as 0.01 mV. However, as only ten traces were averaged to determine connectivity, we might have missed connections with very low release probability.

#### Analysis and statistics

To evaluate correlations in synaptic connection strength, Pearson's *R* is calculated using the following standard formula:



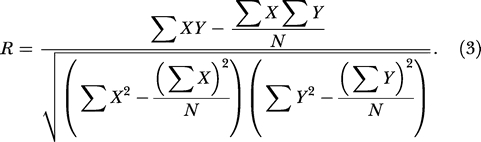



where *X* and *Y* are vectors of paired samples and *N* is the total number of pairs. The *p-*value score of significance is calculated based on Fisher's *z*-score calculated from *R.* For synaptic connection strengths of reciprocally connected pairs, assignment of connection strengths as *X* and *Y* would be arbitrary. Therefore, the *R* value calculated should not depend on the assignment. We use each pair of *X, Y* values twice when calculating the *R* score. For each pair *X_i_, Y_i_,* the pair constructed by flipping the order also entered in the formula; therefore, *N* is twice the total number of pairs. When calculating the *p*-value, the number *N* is taken to be the total number of pairs instead of twice the total number, so as not to overestimate the significance. The correlation scores and *p*-values calculated this way agree with those calculated from a reshuffling procedure by randomly assigning each neuron as either *X* or *Y* and using each pair only once.

To analyze the distribution of synaptic connection strength, we generated fits to the data, by using a mean-square error-based procedure from the MATLAB curve-fitting toolbox in the log–log space. To test whether the experimental distribution has a longer tail than the exponential or Gaussian distribution, we chose a threshold *T,* and counted the number of experimental observations with higher value than *T,* and denoted it by *n,* out of a total of *N* observations. We then calculated the *p*-value as the probability of generating more than *n* observations with values larger than *T* out of *N* observations from the null distributions.

To assess the monotonicity in Figures 7 and 8, we used the Kolmogorov-Smirnov test. For each threshold of synaptic connection strength, we generated an ensemble of 1,000 random matrix sets with matched connection statistics as described in the Motif finding section below. We then computed the distribution of ratios between occurrence counts in the random ensemble and the observed occurrence counts for motif(s) of interest. These ratios are the inverses of the ratios plotted in Figures 2 and 4 in order to avoid division by zero. We then tested for monotonicity between successive pairs of distributions with the Kolmogorov-Smirnov test.

To generate bootstrap distributions for a dataset with *N* observations, we drew an ensemble of 1,000 trials of *N* samples each from the dataset with replacement and computed the appropriate statistic on each trial. The statistics from these trials formed the bootstrap distribution. Mean and standard deviations were then computed on the bootstrap distribution of the chosen statistic.

#### Motif finding

To find overrepresented motifs, we used a statistic based on how the observed counts compare with the expected counts from the null hypothesis. For the null hypothesis, we generated B = 1,000 sets of random connection matrices. Each set contained as many matrices as the number of quadruplets in experimental data. The randomization procedure is as follows: In the two-neuron case, the probability that neuron A is connected with neuron B is the same as experimentally measured, and the connection from B to A is treated independently. In the three-neuron case, each neuron pair is treated as one unit, and the probabilities of having one-way and bidirectional connections within the pair are the same as measured. But how the three pairs form a triplet is random. In the four-neuron case, a 90 × 90 matrix of connections was generated. The fractional counts for each triplet motif to total triplet counts from this big matrix were matched to experiment data using a simulated annealing procedure (see [36]). To generate each random connection matrix in each of the B = 1,000 sets of matrices, we randomly picked four neurons from this 90 × 90 random connection matrix to form a 4 × 4 random connection matrix. This procedure matches the probability of observing a triplet motif to experimental data while randomizing how triplets combine to form quadruplets.

For each motif, we counted the number of its occurrences in measured data and in each set of random matrices. The *p*-value for this motif is the fraction of random matrices with occurrence counts above or below the observed occurrence counts. This tests for significant deviation from random, including both overrepresentation and underrepresentation.

Since we are testing for many motifs simultaneously, we applied the step-down min-P*-*based algorithm for multiple-hypothesis correction [70,71,72]. This procedure ensures weak control for the familywise error rate, which is defined as the probability of at least one type I error (stating that a pattern is overrepresented when it is not) among the family of hypotheses (all motifs). Weak control refers to the fact that type I error is controlled under the complete null hypothesis when all the null hypotheses are assumed to be false. Strong control, which is not used here, would control type I error rate under any combination of true and false null hypotheses, but is harder to achieve. The idea behind the step-down procedures is to order hypotheses according to the raw *p*-values in ascending order. Then for a chosen cutoff *p*-value, the hypotheses are considered successively. For each hypothesis, we test for the possibility of committing at least one type I error for the subset of hypotheses with lower or equal raw *p*-values. Further tests depend on the outcomes of earlier ones. As soon as one fails to reject a null hypothesis, no further hypotheses are rejected. The real procedure combines the testing for all cutoff *p*-values into one procedure, as described in more detail below.

First, we test for *M* motifs with an ensemble *R* of *B* random matrix sets, *R* = {*R_b_,b* ∈ {1,…,*B*}} generated as described above. For each motif *i* ∈ {1,…,*M*}, we calculate the mean occurrence counts over the ensemble and denote it with c¯^*R*^_*i*_ (step 1). Second, we calculate the raw *p*-values p^*^_i_ for each motif *i* for the two-sided statistic *T,* defined as the absolute value of the difference between the observed counts *c* and the mean ensemble counts, *T* = |*c* − c¯^*R*^_*i*_| . We calculate the proportion of sets of random matrices in the ensemble with a larger or equal value for statistic *T* than observed.



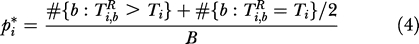



for *i* = 1,…, *M* (step 2). Third, we then order the raw *p*-values such that p^*^_k_1__ >p^*^_k_2__ > … >p^*^_k_M__ (step 3). Fourth, for each *R_b_,b* ∈ {1,…,*B*} and each motif *k_i_,i* ∈ {1,…,*M*}, we repeat step 4: count its number of occurrences *c_k_i_,b_* in R_b_, calculate *T* = |*c_k_i_,b_* − c¯^*R*^_*i*_| and the *p*-value, *p_k_i_,b,_* as in step 2, and then compute *q_k_i_,B_* = min_*l=k_1_,…,k_i_*_*p_l,b_*, , the successive minima of the raw *p*-values (step4). Fifth, the corrected *p*-values *p˜_k_1__* are estimated by calculating the proportion of sets of random matrices in the ensemble in which *q_k_i_,b_* is smaller than or equal to the observed *p*-value p^*^_k_1__ .



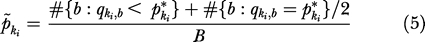



for *i* = 1,…,*M* (step 5). Finally, we enforce the monotonicity constraints by successively setting *p˜_k_i__* to max(*p˜^k_i−1_,^p˜_k_i__* for *i* = 2,…,*M* (step 6).

#### Statistical reconstruction of the network

To generate Figure 9, links were assigned randomly among 50 nodes with the experimentally measured probability of unidirectional and bidirectional connections. Strengths of connections were drawn from the experimentally measured distribution. Then we manually adjusted the connections to have roughly similar probability of occurrence of three-neuron motifs. In constructing this diagram, we assumed that each individual cell has the same distribution of strong and weak synaptic connections. This assumption could be violated if some cells have many stronger synaptic connections while others have few or none. Whether this is the case should be investigated in future studies. This figure is for illustration purposes only.

#### Positions of recorded neurons

To investigate the dependence of connectivity on pairwise distances, we measured the relative coordinates of the recorded cells from slices prepared by biocytin histochemistry after recordings. Distances were not corrected for tissue shrinkage. Since we were most interested in the relationship between connectivity and distance, an equal amount of shrinkage for all slices would not affect our results. Some inhomogeneities of shrinkage were likely, but we did not expect the shrinkage factor to vary greatly across slices.

During recording sessions, the approximate relative positions of cells and the positions of recorded quadruplets in the slice were kept in notes. In most cases, these drawings allowed unambiguous identification of recorded cells, and cell positions were then measured on those cases after identification of the recorded cells. If a quadruplet was totally unconnected, drawings were not provided. However, totally unconnected cells did not have to be identified, and the assignment was made randomly. In some cases, some of the cells in the quadruplet were not well stained. If positions of at least three cells out of the quadruplet could be recovered, the positions of those cells were recorded.

We defined the position of each cell as the three-dimensional coordinate of the axonal initial segment and measured it using the Neurolucida system (MicroBrightField, Williston, Vermont, United States). We estimate the measurement error to be less than 2 μm in *X* and *Y* positions and less than 3 μm in *Z* positions, based on repeated measurements of the same quadruplets. In about 10% of the cases, the initial segment of the axon was obscured by other cells and could not be positively identified, and the cell position was instead measured from the middle of the base of the cell body. In these cases, the measurement error could be as large as 3 μm in *X* and *Y* and 5 μm in *Z.* For each slice, we also estimated the average position of the main apical dendrites of the quadruplet around 300 μm away. The positive direction for the vector from the mean positions of the cells to the estimated average position of the main apical dendrites was defined to be the *pia* direction in the slice. We rotated the original relative coordinates of pairs in the *X, Y* plane so this vector pointed in the positive *Y* direction. We normalized the vector and defined it as the original coordinates of the new unit *Y* vector. The new unit *X* vector is the normal direction to the unit *Y* vector. To calculate the relative *X, Y* coordinates of two cells in the new coordinates, we took the dot product of the relative vector calculated in the original coordinates and the unit *X* and *Y* vectors, also defined in the original coordinates. The relative *Z* coordinates were not subject to rotation. Notice that rotation was done on the relative positions of any two cells and not on the positions of each individual cell.

A total of 817 cells in 83 triplets and 142 quadruplets were measured, resulting in a total of 2,202 possible connections. For each possible connection, the relative position of the target neuron to the originating neuron was plotted in Figure 3A. If a connection was present and was involved in a bidirectional connection, the position was indicated with red. If a connection was present, and the reciprocal connection was not present, the position was indicated with blue. If a connection was not present, regardless of the status of the reciprocal connection, the position was indicated with green. Most of the cells included in the dataset came from nearby positions (<50 μm, 82% of pairs), with the remaining 18% of the connections in the 50 μm–110 μm range (Figure 3B). The densities of red, green, and blue connections are proportional to each other regardless of the distance from the soma. The connection probability is mostly uniform within the range of three-dimensional distances recorded in the experiments (Figure 3C). However, the connection probability for all distances (0.013) is slightly higher than that calculated for the entire dataset (0.0116). This is likely due to less efficient recovery of unconnected quadruplets, as less care was taken to preserve them. The connection strengths do not vary with distance systematically (data not shown), although the distance does seem to have an effect (*p* = 0.02, one-way analysis of variance).

To control for cutting artifacts, we have measured the closest cut ending of the main axons out of the four cells in the quadruplet to the mean position of the cell bodies. The main axons go toward white matter to innervate subcortical structures. When the distance is small, then we might have cut off more portions of the axonal arbor, and cutting artifact might be a concern. However, since the main axons start branching approximately 100 μm from the cell bodies [73], the local connectivity might not be greatly affected (see Figure S3).

We also measured the *Z* coordinate of the slice surface. From this coordinate and the coordinate of the cells, we can deduce the depth of each cell from the slice surface. Cells closer to the surface might have had larger portions of their axonal arbors cut off, which would reduce their connectivity. However, the connectivity seems to be fairly uniform regardless of the depth of either the originating cell or the target cell (see Figure S3). The caveat is that the measurements we have taken from the fixed slices are uncorrected for shrinkage, and differential shrinkage in the *Z* direction might have randomized a trend that might otherwise be present.

## Supporting Information

Appendix S1 Properties of the Lognormal Distribution (39 KB DOC).

Figure S1 Connectivity and Mean Synaptic Strengths of the Connections Are Uniform for All Distances between a Pair of Neurons For the x-axis, positive means the receiving cell is to the right of the sending cell in the slice. For the y-axis, positive means the receiving cell is above the sending cell. For the z-axis, positive means the receiving cell is on top of the sending cell.
(A, D, G, and J) Connection probability for *X, Y,* and *Z* and angle between the vector connecting two neurons and the x-axis separately (no significant variation; all chi square tests, *p* > 0.05). Error bars are 95% confidence intervals estimated from binomial distributions.
(B, E ,H , and K) Mean synaptic strengths for *X, Y,* and *Z* and angle separately (no significant variation; all one-way ANOVA tests, *p* > 0.05). Error bars are standard errors of the mean.
(C, F, I, and L) Histogram of connections for *X, Y,* and *Z* and angle separately.
(363 KB DOC).

Figure S2 The Overrepresentation of Bidirectionally Connected Pairs Is Not Due to Inhomogeneous Connection Probabilities for Neurons of Different Distances Counts relative to random are shown for neurons of different distances. The red line indicates the value calculated for pooled data for neurons of all distances. Notice that the values calculated for certain distances are very similar to that calculated for pooled data. However, the ratio calculated for all distances (3.0) is a bit lower than that calculated for the entire dataset (4.0), probably owing to increased connection probability (0.013 versus 0.0116) caused possibly by inefficient recovery of totally unconnected quadruplets. Error bars are standard deviations from bootstrap.
(119 KB DOC).

Figure S3 Connection Probability and Mean Synaptic Connection Strengths Are Not Greatly Modified by Cutting (A) Connection probability is uniform with regard to the distance to the closest main axon cut ending (*p* = 0.077, chi square test). Notable exception is distances more than 600 μm away, where the connection probability seems to be slightly increased. However, since there are relatively few neurons with axon cut distance of more than 600 μm and the increase in connection probability is not statistically significant, we do not expect this to fully explain our results.
(B) Mean synaptic connection strength does not vary systematically with regard to the distance to the closest main axon cut ending (however, mean strength depends on distance; *p* = 0.02 by one-way ANOVA).
(C) Histogram of neurons with certain axon cut distances.
(D) Connection probability is uniform with regard to the depth of both the neuron sending the connection and the neuron receiving the connection (*p* = 0.99, chi square test).
(E) Mean synaptic connection is uniform with regard to the depth of both the neuron sending the connection and the neuron receiving the connection (*p* = 0.2, one-way ANOVA).
(F) Histogram of recorded neurons with certain depth.
Error bars in (A) and (D) are 95% confidence intervals estimated from binomial distribution. Error bars in (B) and (E) are standard errors of the mean.
(244 KB DOC).

Figure S4 EPSP Size and Rate of Connectivity Does Not Significantly Depend on Animal Age (A and B) We found no statistically significant difference among EPSP amplitudes for animals of different ages (one-way ANOVA in log space, *p* = 0.36). We note, however, that there is a weak downward trend, in agreement with the observation of [34] that L5-to-L5 synaptic strength is significantly weaker in P28 animals than in P14 animals. Error bars are standard errors of the mean.
(C) The connectivity rate does not depend on animal age (chi square test, *p* = 0.92). Error bars are 95% confidence intervals calculated from binomial distribution.
(218 KB DOC).

Figure S5 Main Results of This Paper Are Still Valid for the Subset of Data from P14–P16 Animals (A and B) Overrepresentation of bidirectional connections and highly connected triplets. Numbers on top of bars are actual counts.
(C) Synaptic connection strengths are well fit by the lognormal distribution. Number on top of dots are actual counts (not shown when greater than 50).
(D) Probability of significant deviation from random for a given triplet motif.
(E and F) Increase in overrepresentation of bidirectional connections and highly connected triplets for increasingly higher connection strength thresholds.
(847 KB DOC).

Figure S6 Quadruplet Catalogue (1.2 MB DOC).

Figure S7 Synaptic Strengths of Incoming and Outgoing Connections Are Weakly Correlated (A) Scatter plot of incoming synaptic connection strength. A weak correlation of 0.2 is observed (*p* = 0.029).
(B) Scatter plot of outgoing synaptic connection strength. A weak correlation of 0.17 is observed (*p* = 0.054).
(C) Scatter plot of outgoing and incoming synaptic connection strength. A weak correlation of 0.13 is observed (*p* = 0.039). All correlations calculated using Pearson's *R* method in log space.
(231 KB DOC).

Figure S8 EPSP Standard Deviation Depends Weakly on EPSP Amplitude (A) EPSP standard deviation depends weakly on EPSP amplitude.
(B) Coefficient of variation is inversely proportional to the EPSP amplitude. Note the log–log scale.
(128 KB DOC).

Table S1 Quadruplet Counts Quadruplets are numbered according to the catalogue in Figure S1.
(124 KB DOC).

## Abbreviations

EPSP: excitatory postsynaptic potential
LTP: long-term potentiation
