# Correction: Highly Nonrandom Features of Synaptic Connectivity in Local Cortical Circuits

**DOI:** 10.1371/journal.pbio.0030350

**Published:** 2005-10-11

**Authors:** Sen Song, Per Jesper Sjöström, Markus Reigl, Sacha Nelson, Dmitri B Chklovskii

In *PLoS Biology*, volume 3, issue 3: DOI: 10.1371/journal.pbio.0030068

In the caption for Figure 5A, the formula for the lognormal fit was given as follows:



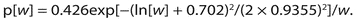



.

The second squaring exponent should have been inside the parentheses, so it reads as follows:



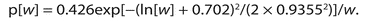



.

