# Supplementary material for: Highly Nonrandom Features of Synaptic Connectivity in Local Cortical Circuits
Source: PLoS Biol. 2005 Mar 1;3(3):e68. doi: 10.1371/journal.pbio.0030068 (PMC1054880; doi:10.1371/journal.pbio.0030068)
Supplement: Supplementary file 1 [file pbio.0030068.sd001.doc]

Appendix S1. Properties of the lognormal distribution.

A random variable *X* has a lognormal distribution if the random variable *Y* = ln *X* has a normal (i.e., Gaussian) distribution. We denote the distribution of *X* by *p(x)*. If the associated normal distribution has mean µ and variance σ^2^, then the lognormal distribution has distribution function *p*[*w*] = Aexp[−(ln[*w*] -µ)^2^/2σ^2^]/*w,* where A is a normalization constant*.* It is skewed, with mean$e^{^{\mu+\sigma^{2}/2}}$, median$e^{\mu}$**,** and mode $e^{\mu-\sigma^{2}}$(most probable value, peak in the distribution curve). For the synaptic connection strength curve in this paper, the associated normal distribution has mean µ=-0.702mV, and variance σ^2^=0.8752mV^2^. The mean, median, and mode of the lognormal distribution are respectively 0.77mV, 0.50mV, and 0.21mV. The lognormal distribution is similar in shape to power-law distributions for a wide range of values. When plotted in log–log scale the lognormal curve is close to linear in the “waist” region (see Figure 5A). The curve would be linear for a power-law distribution. This can be understood by writing

For $e^{-(\sigma^{2}-\mu)}<x<e^{(\sigma^{2}-\mu)}$, which in the case of the synaptic connection strength distribution is 0.21 mV < *x* < 4.76 mV; contribution of the quadratic term is smaller than half of the linear term, and the lognormal distribution looks linear [74]. Because we do not have many data points for large synapses with large connection strengths, it is possible that the tail of the distribution is better fit by a power law, although the lognormal distribution does provide a quite good fit to the whole dataset.

One useful additional property of the lognormal distribution is that the product of lognormally distributed variables is again lognormally distributed. Therefore, if we divide the synaptic strength on one day by the synaptic strength on another day, the distribution of fractional changes again obeys a lognormal distribution.

The lognormal distribution is extensively discussed in the ecology literature. The number of members of an individual species is observed to obey a lognormal distribution. The stochastic Gompertz growth model is often used to explain this phenomenon [56,59]. In this model, the following stochastic differential equation is assumed:

where *N(t)* is a time-dependent random variable denoting the distribution of number of members of a particular species, and *t* is time.$\overline{N}$is the mean number of members at equilibrium. *W(t)* is a Wiener process (zero mean and unit variance) and models the random fluctuations in the amount of growth over time, and σ is the standard deviation of the fluctuations. The equilibrium distribution of *N* becomes lognormal:

,

where A is a constant to normalize the distribution function. An analogous growth equation could be used to model the synaptic connection strength studied in this paper.
