# Supplementary material for: Highly Nonrandom Features of Synaptic Connectivity in Local Cortical Circuits
Source: PLoS Biol. 2005 Mar 1;3(3):e68. doi: 10.1371/journal.pbio.0030068 (PMC1054880; doi:10.1371/journal.pbio.0030068)
Supplement: Supplementary file 2 [file pbio.0030068.sg001.doc]

**Figure S1.** Connectivity and mean synaptic strengths of the connections are uniform for all distances between a pair of neurons. For the X axis, positive means the receiving cell is to the right the sending cell in the slice. For the Y axis, positive means the receiving cell is above of the sending cell. For the Z axis, positive means the receiving cell is on top of the sending cell.

**(A,D,G,J)** Connection probability for X,Y,Z and angle between the vector connecting two neurons and the X axis separately (No significant variation, all chi square tests p>0.05). Error bars are 95% confidence intervals estimated from binomial distributions.

(**B,E,H,K)** Mean synaptic strengths for X,Y,Z and angle separately (No significant variation, all one-way ANOVA tests p>0.05). Error bars are standard errors of the mean.

(**C,F,I,L)** Histogram of connections for X,Y,Z and angle separately.
