# Supplementary material for: Highly Nonrandom Features of Synaptic Connectivity in Local Cortical Circuits
Source: PLoS Biol. 2005 Mar 1;3(3):e68. doi: 10.1371/journal.pbio.0030068 (PMC1054880; doi:10.1371/journal.pbio.0030068)
Supplement: Supplementary file 3 [file pbio.0030068.sg002.doc]

**Figure S2.** The overrepresentation of bi-directionally connected pairs is not due to inhomogeneous connection probabilities for neurons of different distances. Counts relative to random are shown for neurons of different distances. The red line indicates the value calculated for pooled data for neurons of all distances. Notice that the values calculated for certain distances are very similar to that calculated for pooled data. However, the ratio (3.0) calculated for all distances is a bit lower than that calculated for the entire dataset(4.0), probably due to increased connection probability (0.013 versus 0.0116) caused possibly by inefficient recovery of totally unconnected quadruplets. Error bars are standard deviations from bootstrap.
