# Supplementary material for: Highly Nonrandom Features of Synaptic Connectivity in Local Cortical Circuits
Source: PLoS Biol. 2005 Mar 1;3(3):e68. doi: 10.1371/journal.pbio.0030068 (PMC1054880; doi:10.1371/journal.pbio.0030068)
Supplement: Supplementary file 4 [file pbio.0030068.sg003.doc]

**Figure S3.** Connection probability and mean synaptic connection strengths are not greatly modified by cutting.

(**A)** Connection probability is uniform with regard to the distance to the closest main axon cut ending (p=0.077 by chi square test). Notable exception is distances >600μm away, where the connection probability seems to be slightly increased. However, since there are relatively few neurons with axon cut distance of more than 600μm and the increase in connection probability is not statistically significant, we do not expect this to fully explain our results.

(**B)** Mean synaptic connection strength does not vary systematically with regard to the distance to the closest main axon cut ending (However, mean strength depends on distance, p=0.02 by one-way ANOVA).

(**C)** Histogram of neurons with certain axon cut distances.

(**D)** Connection probability is uniform with regard to the depth of both the neuron sending the connection and the neuron receiving the connection (p= 0.99 by chi-square test)

**(E)** Mean synaptic connection is uniform with regard to the depth of both the neuron sending the connection and the neuron receiving the connection (p=0.2 by one-way ANOVA)

(**F)**Histogram of recorded neurons with certain depth.

Error bars in **A** and **D** are 95% confidence intervals estimated from binomial distribution. Error bars in **B** and **E** are standard errors of the mean.
