# Supplementary material for: Highly Nonrandom Features of Synaptic Connectivity in Local Cortical Circuits
Source: PLoS Biol. 2005 Mar 1;3(3):e68. doi: 10.1371/journal.pbio.0030068 (PMC1054880; doi:10.1371/journal.pbio.0030068)
Supplement: Supplementary file 5 [file pbio.0030068.sg004.doc]

**Figure S4**: EPSP size and rate of connectivity does not significantly depend on animal age.

(**A,B)** No statistically significant difference among EPSP amplitudes for animals with different ages was found (one way ANOVA in log space, p=0.36). We note, however, that there is a weak downward trend, in agreement with the observation of [34] that L5-to-L5 synaptic strength in P28 animals is significantly weaker than in P14 animals. Error bars are standard errors of the mean. (**C.**)The connectivity rate does not depend on animal age (chi-square test, p=0.92). Error bars are 95% confidence intervals calculated from binomial distribution.
