# Supplementary material for: Highly Nonrandom Features of Synaptic Connectivity in Local Cortical Circuits
Source: PLoS Biol. 2005 Mar 1;3(3):e68. doi: 10.1371/journal.pbio.0030068 (PMC1054880; doi:10.1371/journal.pbio.0030068)
Supplement: Supplementary file 6 [file pbio.0030068.sg005.doc]

**Figure S5**: Main results of this paper are still valid for the subset of data from P14-16 days animals

(**A**,**B)** Overrepresentation of bidirectional connections and highly connected triplets. Numbers on top of bars are actual counts.

(**C)** Synaptic connection strengths are well fit by the lognormal distribution**.** Number on top of dots are actual counts (not shown when >50).

(**D)** Probability of significant deviation from random for a given triplet motif.

(**E,F)** Increase in overrepresentation of bidirectional connections and highly connected triplets for increasingly higher connection strength thresholds.
