# Supplementary material for: Highly Nonrandom Features of Synaptic Connectivity in Local Cortical Circuits
Source: PLoS Biol. 2005 Mar 1;3(3):e68. doi: 10.1371/journal.pbio.0030068 (PMC1054880; doi:10.1371/journal.pbio.0030068)
Supplement: Supplementary file 8 [file pbio.0030068.sg007.doc]

**Figure S7** Synaptic strengths of incoming and outgoing connections are weakly correlated.

**(A)** Scatter plot of incoming synaptic connection strength. A weak correlation of 0.2 is observed (*p*=0.029).

(**B)** Scatter plot of outgoing synaptic connection strength. A weak correlation of 0.17 is observed (*p*=0.054).

(**C)** Scatter plot of outgoing and incoming synaptic connection strength. A weak correlation of 0.13 is observed (*p*=0.039). All correlations calculated using Pearson’s *R* method in log space.
