# Supplementary material for: Highly Nonrandom Features of Synaptic Connectivity in Local Cortical Circuits
Source: PLoS Biol. 2005 Mar 1;3(3):e68. doi: 10.1371/journal.pbio.0030068 (PMC1054880; doi:10.1371/journal.pbio.0030068)
Supplement: Supplementary file 9 [file pbio.0030068.sg008.doc]

Figure S8 EPSP standard deviation depends weakly on EPSP amplitude

(A) EPSP standard deviation depends weakly on EPSP amplitude

(B) Coefficient of variation is inversely proportional to the EPSP amplitude. Note the log-log scale.
