# Supplementary material for: Highly Nonrandom Features of Synaptic Connectivity in Local Cortical Circuits
Source: PLoS Biol. 2005 Mar 1;3(3):e68. doi: 10.1371/journal.pbio.0030068 (PMC1054880; doi:10.1371/journal.pbio.0030068)
Supplement: Supplementary file 10 [file pbio.0030068.st001.doc]

# Supporting Materials

**Table S1.** Quadruplet counts.

Quadruplets are numbered according to the catalogue in Figure S6.
